# Female Representation and Quality Indicators in Anesthesiology and Critical Care Podcasts

**DOI:** 10.1111/aas.70356

**Published:** 2026-10-14

**Authors:** Perla Steinsdóttir, Sana Asif, Rakel N. Kristinsdóttir, Alexander Rosenström, Andrea Niklasson, Nicholas Dietz, Siri Å. Fåhraeus, Johan Hylander, Martin F. Bjurström

**Affiliations:** ^1^ Department of Surgical Sciences, Anaesthesiology and Intensive Care Medicine Uppsala University Uppsala Sweden; ^2^ Department of Surgical Sciences, Clinical Pain Research Uppsala University Uppsala Sweden; ^3^ Department of Neurosurgery University of Louisville Louisville Kentucky USA; ^4^ Centre for Disaster Medicine, Department of Diagnostics and Intervention Umeå University Umeå Sweden

**Keywords:** anesthesiology, critical care, female representation, medical education, podcast

## Abstract

**Background:**

Podcasts have emerged as prominent tools for asynchronous medical education, widely adopted across training levels and specialties. Diverse representation strengthens medical discourse, making an evaluation of speaker demographics essential for understanding equity and quality in digital knowledge dissemination. This study systematically evaluates female representation and quality assurance indicators in anesthesiology and critical care podcasts, including longitudinal publication trends.

**Methods:**

We systematically searched Apple Podcasts and Spotify using specialty‐specific terms and manually reviewed the top twenty journals in the field for affiliated podcasts. Included were active, English‐language, physician‐hosted podcasts.

**Results:**

Eighty‐five podcasts (53.0% US‐based) comprising 10,190 episodes were analyzed. Of 12,522 hosts, 31.5% (*n* = 3945) were female; of 10,822 guests, 35.9% (*n* = 3882) were female. Notably, among US‐based podcasts, female guest representation exceeded the 26.5% female workforce proportion among US anesthesiologists. Episodes with exclusively male hosts had 2.13 times the odds of featuring only male guests (95% CI 1.88–2.41). A positive trend of increasing female participation was observed from 2010 to 2024. Content support was variable: using broad, inclusive definitions, 42.0% of episodes had show notes and 48.4% provided supplemental material. Analysis of quality‐related structures showed that 40.0% (*n* = 34) of podcasts produced episodes linked to a publication, 50.6% featured some episodes with three or more speakers, 21.2% of podcasts were journal‐affiliated, and 20.0% were university‐affiliated.

**Conclusion:**

While a sex imbalance persists in anesthesiology and critical care podcasting, female participation among US‐based podcasts exceeds local workforce demographic benchmarks, with a clear positive trend from 2010 to 2024. However, persistent sex similarity in speaker selection suggests ongoing biases. Quality assurance mechanisms remain highly variable, highlighting opportunities for incremental improvement. Future efforts could focus on structured guest selection and transparent quality indicators to accelerate progress toward equitable representation and educational quality in this influential medium.

**Editorial Comment:**

In modern times, many clinicians look for, and listen to, podcasts aimed at education in clinical specialties. This sampling of podcasts related to our specialty looked at how females and males contributed to podcast content generation and dissemination, as a marker for male versus female representation in speakership for continuing specialty education.

## 1 Introduction

Podcasts have become a mainstream educational tool in medicine, with dedicated content for most specialties [1, 2]. The integration of podcasts into postgraduate training is significant: a majority of anesthesia residents use them, emergency medicine residents report more time listening to podcasts than reading textbooks, and internal medicine residents find podcasts more helpful than traditional resources [3, 4].

A notable feature of this medium is the apparent sex disparity among speakers. In popular medical podcasts, male voices are predominant as hosts and guests. For example, women constituted only 16.2% of speakers on top emergency medicine podcasts, despite representing 30% of US emergency physicians [5]. This imbalance may influence engagement, as male sex has been predictive of podcast use among anesthesiologists [6].

While sex disparities in medical podcasting have been documented in fields like emergency medicine, anesthesiology represents a uniquely instructive context. With female physicians comprising only 26.5% of the US anesthesiology workforce, one of the lowest proportions among specialty fields [7], the specialty faces challenges in recruitment and retention of women [8]. Moreover, almost 4 out of 10 anesthesia residents use podcasts on a weekly basis as a primary educational resource [6, 9]. Given that podcasts often model clinical reasoning and technical skills, the sex composition of speakers may subtly shape trainees' perceptions of who belongs in the specialty.

We conceptualize sex representation in medical podcasts through three complementary frameworks: first, as an equity outcome that should reasonably reflect the demographic composition of the specialty; second, as a marker of diversity, whereby the inclusion of diverse voices enriches clinical discourse and challenges dominant perspectives; and third, as a contributor to educational quality, given that exposure to diverse role models influences trainee engagement, specialty choice, and retention. We hypothesize that the underrepresentation of female voices may reduce the appeal of podcasts to female clinicians and trainees, potentially perpetuating a feedback loop of lower engagement and reduced representation.

Yet, no study has systematically examined sex distribution among hosts and guests in anesthesiology and critical care podcasts. Furthermore, robust quality assurance frameworks are lacking. This study aims to characterize female representation and quality assurance processes in anesthesiology and critical care podcasts, proposing that greater transparency and diversity could improve the utility of this important asynchronous educational medium.

## 2 Methods

### 2.1 Search Strategy

A systematic search was conducted on two leading platforms, Apple Podcasts and Spotify, in March 2025. We used individual search terms: anesthesia, anaesthesia, anesthesiology, anaesthesiology, critical care, intensive care, and ICU. Additionally, the websites of the top 10 journals by impact factor in anesthesiology and the top 10 in critical care (20 journals total) were manually reviewed. Preliminary searches on podcast platforms presented methodological challenges: unlike academic databases that return unique citations, these platforms return individual episode‐level results, generating excessive duplicate and irrelevant entries for a single search term. A conventional PRISMA‐based systematic review, which relies on deduplication and unique records, was therefore not feasible. Consequently, the study was not preregistered. Instead, we adopted a systematic search approach, screening podcast‐level results and applying inclusion criteria manually, as detailed below.

### 2.2 Inclusion Criteria

One reviewer (PS) screened podcasts for inclusion. Eligible podcasts were active (> 1 episode in 2024), in English, and focused on anesthesiology and/or critical care. Podcasts without a physician host were excluded to ensure a consistent, manageable scope.

### 2.3 Data Extraction

Using standardized forms, trained reviewers collected data by listening to episodes and reviewing associated show notes and official podcast websites. For each podcast, we recorded its country of origin, host name(s) (if ≤ 2 hosts), establishment year, and subspecialty. For every episode published from the podcast's inception through 2024, we extracted number and sex of hosts, number and sex of guests, episode length, and publication year. The sex of hosts and guests was determined using multiple identifiers: name, voice, pronouns in the episode or show notes (pronouns she/her proxy for female and he/him proxy for male), and faculty website information. Quality indicators, including the presence of show notes and supplemental data, were also documented per episode. Show notes were defined as any type of description of topic(s) presented or discussed, in its most extensive form comprehensive summaries and outlines. Supporting information could range from brief reference lists/article links to infographics, quizzes etc. Moreover, based on a previous attempt to assess peer‐review processes in anesthesiology podcasts, the following data were collected: whether ≥ 1 episode was created in the context of a publication (e.g., discussion centered around a recently published article), grand rounds, had ≥ 3 speakers, and if the podcast was associated with a journal or university [10].

### 2.4 Statistical Analysis

Data analyses were conducted utilizing SPSS version 29.0 (Armonk, NY: IBM Corp.). Descriptive statistics were used to summarize data, mainly as absolute numbers and percentages. A “Male–Female Index” (ranging from 0 to 1) was constructed to analyze temporal trends in sex composition. The index is defined as the proportion of males relative to the total:

$$\text{Male}-\text{female index}=\frac{n\left(\text{males}\right)}{n\left(\text{males}+\text{females}\right)}$$

where 0 represents an entirely female sample and 1 an entirely male sample. Logistic regression models were used to test associations between sex of host(s) and sex of guest(s). A *p* < 0.05 was considered significant.

## 3 Results

### 3.1 Included Podcasts

The search identified 85 relevant podcasts (Figure 1).

**FIGURE 1:**
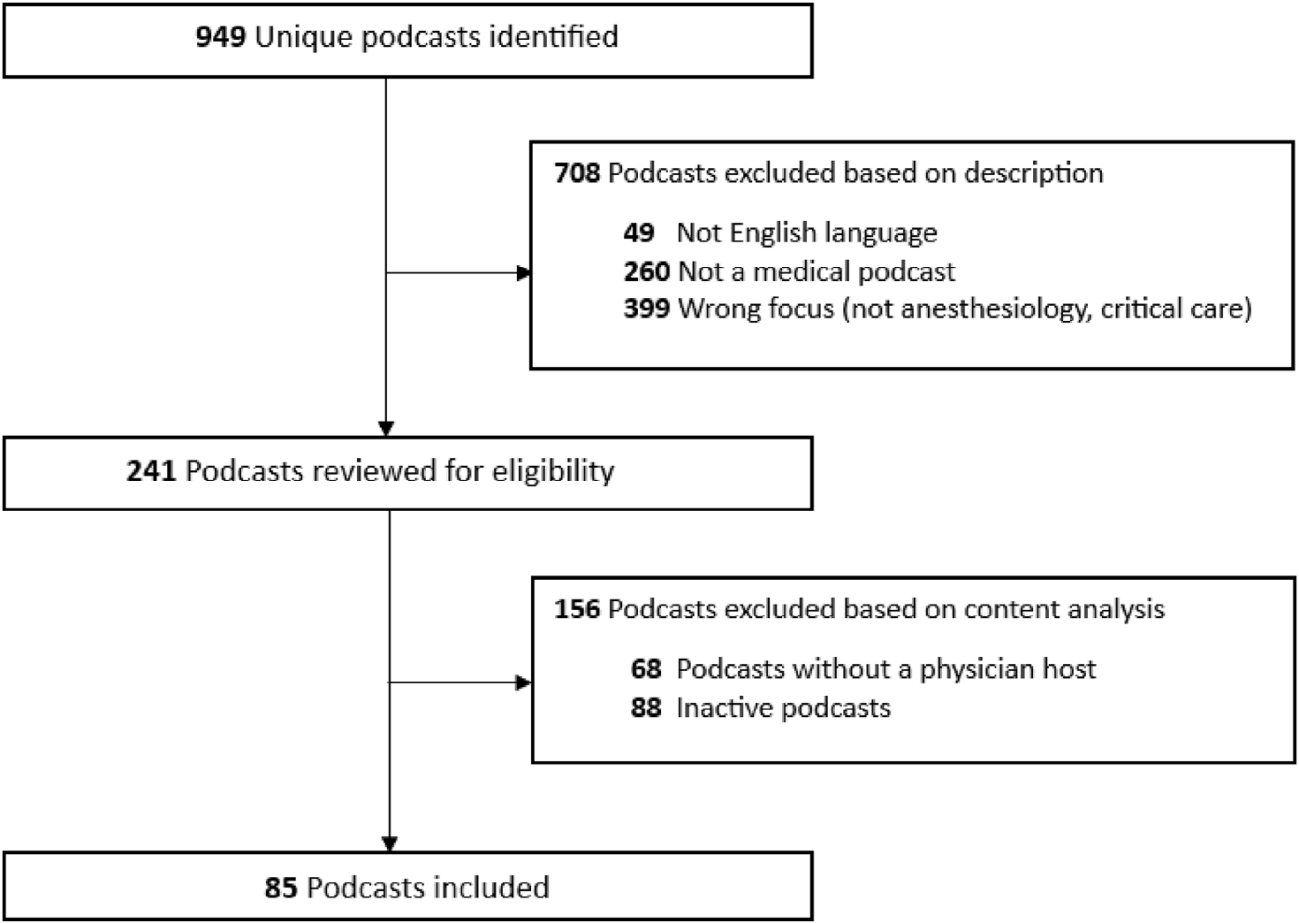
Podcast selection flowchart.

### 3.2 Characteristics of Included Podcasts

An overview of podcast characteristics is provided in Table 1. A total of 10,190 episodes were analyzed. The United States was the most frequent country of origin (53.0%), followed by Australia (17.7%), and Great Britain (14.1%). The oldest podcast in the sample launched in 2005, though most were established in 2019 or later (*n* = 64, 75.3%). The number of episodes per podcast ranged from 2 to 1723. Yearly publication counts demonstrated a steady increase from 2005 to 2024, with notable exceptions in 2008 and the 2020–2022 period (Figure 2). The number of hosts per podcast varied, with most podcasts having 1–2 hosts (*n* = 44, 51.8%). The median episode length was 25 min (interquartile range: 21.0) (Figure 3).

**TABLE 1:** Characteristics of the included anesthesiology and critical care medicine podcasts.

Podcast name	Country of origin	Active since	Subspecialty	Male hosts^a^	Female hosts^a^	Guests^b^	Male guests^b^	Female guests^b^	Average length (min)	Number of episodes published	Show notes^g^	Supple‐mental^h^	Publi‐cation^d^	≥ 3 speakers^e^	Journal^f^	University^c^
ABCs of anaesthesia	AUS	2021	None	2	0	50	30	20	40.6	65	0	29.2	No	No	No	No
Airway, breathing, conversation	CAN	2023	None	1	1	86	41	45	48.8	50	0	0	No	Yes	No	Yes
Anaestheasier	GBR	2022	None	2	1	0	0	0	14.2	80	0	75.0	No	No	No	No
Anaesthesia coffee break	AUS	2020	None	2	0	35	20	15	37.7	76	15.8	38.2	No	Yes	No	No
Anaesthesia on air	GBR	2019	None	38	26	181	93	88	31.0	137	0	46.0	Yes	Yes	No	No
Anesthesia and analgesia podcast	USA	2008	None	35	19	299	203	96	21.0	256	0	100	Yes	Yes	Yes	No
Anesthesia and critical care reviews and commentary (ACCRAC) podcast	USA	2016	None	1	0	311	190	121	48.8	294	32.3	75.5	Yes	Yes	No	Yes
Anesthesia learn on the go	USA	2019	None	27	8	2	0	2	16.0	32	0	0	No	No	No	Yes
Anesthesia patient safety podcast	USA	2020	None	0	1	138	82	56	17.5	234	100	100	Yes	Yes	No	No
Anesthesia STAT: the Stanford anesthesia tutorial podcast	USA	2022	None	1	0	6	5	1	40.6	5	100	0	No	No	No	Yes
Anesthesia unmasked	USA	2024	None	1	0	9	5	4	29.6	5	100	20.0	No	No	No	No
Anesthesia wise guys	USA	2016	None	4	0	100	74	26	48.0	91	0	0	No	Yes	No	No
*Anesthesiology Journal*	USA	2023	None	1	1	85	73	12	23.2	70	0	100	Yes	Yes	Yes	No
Anesthesiology news presents	USA	2019	None	7	3	120	68	52	22.9	52	21.2	59.6	Yes	Yes	No	No
ANZCA	AUS&NZL	2022	None	2	0	5	1	4	30.0	15	0	66.7	No	No	No	No
APA podcasts	GBR	2017	PedA	2	0	23	15	8	15.7	20	0	15.0	Yes	No	No	No
ASRA news	USA	2019	Pain & RA	1	1	2	2	0	11.2	264	0	100	Yes	No	Yes	No
ASRA RAPP	USA	2016	Pain & RA	3	1	178	109	69	39.4	66	0	43.9	Yes	Yes	No	No
Australian anaesthesia	AUS	2020	None	0	1	96	51	45	25.4	96	100	100	Yes	Yes	No	No
Behind the drapes: anesthesiology and critical care medicine	USA	2022	None	1	0	57	30	27	31.8	79	100	0	No	Yes	No	Yes
BJA education podcasts	GBR	2013	None	6	3	116	82	34	28.4	87	0	100	Yes	Yes	Yes	No
Block it like it's hot	GBR&USA	2022	RA	2	0	3	1	2	49.6	24	0	29.2	No	No	No	No
Breathe easy	USA	2015	PMCC	99	93	633	309	324	28.9	431	0	13.5	Yes	Yes	Yes	No
CARE team sessions	GBR	2022	PreCC	1	0	25	18	7	45.3	19	0	0	No	Yes	No	No
Central line by ASA	USA	2019	None	6	3	262	163	99	36.9	150	100	100	Yes	Yes	Yes	No
*CHEST Journal* podcasts	USA	2014	PMCC	13	5	388	284	104	32.0	272	100	2.6	Yes	Yes	Yes	No
Coffee and a gas: a wellbeing podcast	GBR	2021	None	10	23	31	14	17	33.0	25	0	0	No	No	No	No
Count to 10	AUS&NZL	2022	None	1	0	9	7	2	38.0	31	0	0	No	Yes	No	No
CritCare bites	SGP	2024	CC	2	1	5	5	0	28.0	14	92.9	92.9	Yes	No	No	No
Critical care perspectives in emergency medicine	USA	2017	EMCC	4	0	20	14	6	27.2	111	100	36.0	Yes	Yes	No	No
Critical care reviews podcast	IRL	2016	CC	1	0	334	238	96	35.6	135	0	0	Yes	Yes	No	No
Critical care scenarios	USA	2019	CC	2	0	85	57	28	37.2	193	38.3	39.9	Yes	No	No	No
Critical care time	USA	2023	None	2	0	25	19	6	67.4	40	100	100	Yes	Yes	No	No
Critical matters	USA	2019	None	1	0	135	100	35	60.9	145	0	100	No	No	No	No
Deep breaths	AUS	2020	None	0	2	67	50	17	22.7	133	100	100	Yes	Yes	No	No
Depth of anesthesia	USA	2019	None	2	0	46	39	7	35.3	38	100	100	No	No	No	No
eCritCare podcast	AUS	2019	CC	2	0	1	1	0	27.8	123	0	0	Yes	No	No	No
EMCrit FOAM feed	USA	2009	EMCC	1	0	317	264	53	26.5	530	85.5	93.0	Yes	Yes	No	No
ESAIC podcast series on anaesthesia & intensive care	Int.	2022	None	23	14	66	39	27	25.1	49	100	2.0	No	Yes	No	No
ESICM talk	Int.	2021	CC	25	15	115	80	35	20.7	85	100	62.4	No	No	Yes	No
*European Journal of Anaesthesiology*	Int.	2021	None	28	5	36	25	11	20.4	33	0	9.1	No	Yes	Yes	No
FICMLearning	GBR	2020	CC	5	1	83	53	30	22.2	62	0	0	No	No	No	No
Fresh flow podcast	USA	2023	None	2	0	12	9	3	36.3	12	100	0	No	No	No	Yes
GasGasGas—primary frca “fun”	GBR	2024	None	1	0	0	0	0	16.2	19	100	100	No	No	No	No
Going under: anesthesia answered	USA	2024	None	2	0	15	12	3	35.5	30	0	6.7	No	No	No	Yes
ICU doc talk	USA	2022	CCC	1	0	0	0	0	43.**8**	67	0	0	No	No	No	No
ICU educational series	USA	2021	CC	0	2	32	15	17	6.8	30	0	0	No	No	Yes	Yes
ICU fellowship PrepCast	GBR	2021	CC	1	1	1	1	0	24.6	27	0	0	No	No	No	No
ICU primary PrepCast	AUS	2018	None	2	1	0	0	0	17.8	94	95.7	0	No	No	No	Yes
ICUedu	USA	2022	CC	0	1	0	0	0	28.9	53	100	56.6	No	No	No	No
*Indian Journal of Anaesthesia*	IND	2023	None	2	2	78	58	20	46.3	22	59.1	0	No	Yes	Yes	No
Maryland CC project	USA	2013	CC	N/A	N/A	298	230	68	50.8	286	100	100	Yes	Yes	No	Yes
Mastering intensive care	AUS	2017	CC	1	0	105	68	37	64.4	93	100	68.8	No	No	No	No
MECCSIG podcasts	AUS	2020	None	35	10	16	10	6	29.3	23	0	13.0	No	No	No	Yes
Neurocritical care society podcast	USA	2017	NCC	28	14	179	114	65	25.6	150	5.3	59.3	Yes	Yes	No	No
New Zealand anaesthesia	NZL	2022	None	0	1	26	10	16	35.4	18	5.6	16.7	No	No	No	No
NYSORA anesthesia updates	USA	2024	None	1	0	0	0	0	10.5	17	5.9	0	No	No	No	No
Obsgynaecritcare	AUS	2017	OA	1	0	156	114	42	30.4	140	100	100	No	No	No	No
OpenAnesthesia multimedia	USA	2009	None	41	33	678	401	277	22.1	932	0.8	10.3	Yes	Yes	Yes	No
Optimal anesthesia	IND	2023	None	1	0	0	0	0	3.0	2	0	0	No	No	No	No
PCICS podcast	USA	2017	PedCCC	11	18	243	76	167	32.2	121	0	14.0	No	Yes	No	No
Pediatric anesthesia podcast	Int.	2021	PedA	2	1	41	22	19	17.2	27	0	96.3	No	No	Yes	No
Pediatrica intensiva	Int.	2020	PedCC	2	2	35	16	19	57.5	24	20.8	70.8	No	Yes	No	No
Perioperative medicine podcast series	AUS	2018	None	3	1	57	31	26	21.8	50	100	0	No	No	No	Yes
PulmPEEPs	USA	2021	PMCC	6	4	161	73	88	38.4	92	78.3	78.3	Yes	Yes	No	No
Rapid sequence education	AUS	2023	None	3	0	0	0	0	33.0	10	100	0	No	No	No	No
Rapid sequence: *The Anaesthesia Journal* podcast	GBR	2020	None	47	32	185	114	71	31.0	73	0	100	Yes	Yes	Yes	No
RAPM focus	USA	2022	Pain & RA	3	0	52	38	14	34.9	34	100	100	No	No	Yes	No
Residents in a room by American society of anesthesiologists	USA	2019	None	38	31	179	105	74	33.5	71	0	0	No	Yes	No	No
ResusX:Podcast	USA	2023	CC	5	1	115	87	28	15.7	114	99.1	0	Yes	Yes	No	No
Saving lives podcast: critical care w/eddyjoemd	USA	2020	CC	1	0	1	1	0	11.1	144	87.5	86.1	Yes	No	No	No
SCCM podcast	USA	2005	CC	14	7	574	404	170	26.1	541	0.6	11.5	Yes	Yes	No	No
Sevo sistas	USA	2022	None	0	1	58	11	47	19.8	93	100	0	No	No	No	No
SNACC podcast	USA	2015	NeurA & CC	8	12	57	40	17	18.3	65	0	0	Yes	Yes	Yes	No
So you need an operation—patient information podcast series	AUS	2024	None	1	1	19	6	13	19.2	10	0	0	No	Yes	No	Yes
Society for head and neck anesthesia	USA	2023	HNA	1	0	4	3	1	20.2	4	0	0	No	No	Yes	No
Style points	USA	2023	None	2	0	30	17	13	47.0	13	0	0	No	No	No	Yes
Super high yield anesthesia: 2nd edition	USA	2022	None	0	0	6	6	0	31.7	30	83.3	90.0	No	No	No	No
Tasty morsels of critical care	IRL	2020	CC	1	0	0	0	0	5.8	85	0	100	No	No	No	No
The critical care commute podcast	CAN	2022	CC	2	0	76	62	14	26.4	68	5.9	45.6	Yes	Yes	No	Yes
The pre‐admission clinic: roads to anaesthetics	AUS	2024	None	0	0	0	0	0	27	3	0	0	No	No	No	No
TopMedTalk	GBR	2017	None	5	2	2646	1784	862	21.6	1723	61.8	50.7	Yes	Yes	No	No
Vital times: the CSA podcast	USA	2022	None	2	1	65	29	36	46.2	33	0	0	No	Yes	No	No
WUA podcast	CAN	2024	None	0	1	5	5	0	10.0	2	100	50	No	Yes	No	Yes
Yale anesthesiology	USA	2023	OA	1	0	32	14	18	36	28	0	100	Yes	Yes	Yes	Yes

Abbreviations: CC, critical care; CCC, cardiac critical care; EMCC, emergency medicine critical care; HNA, head and neck anesthesia; NeurCC, neurocritical care; OA, obstetric anesthesia; PedA, pediatric anesthesia; PedCC, pediatric critical care; PedCCC, pediatric cardiac critical care; PMCC, pulmonary medicine and critical care; PreCC, prehospital critical care; RA, regional anesthesia.

^a^ Number of individual hosts.

^b^ Total number of guests.

^c^ Association with a university.

^d^ Some episodes created in the context of a publication (e.g., discussion about a recently published article).

^e^ Some episodes with ≥ 3 speakers.

^f^ Association with a journal.

^g^ Proportion of episodes with show notes.

^h^ Proportion of episodes with supplemental material.

**FIGURE 2:**
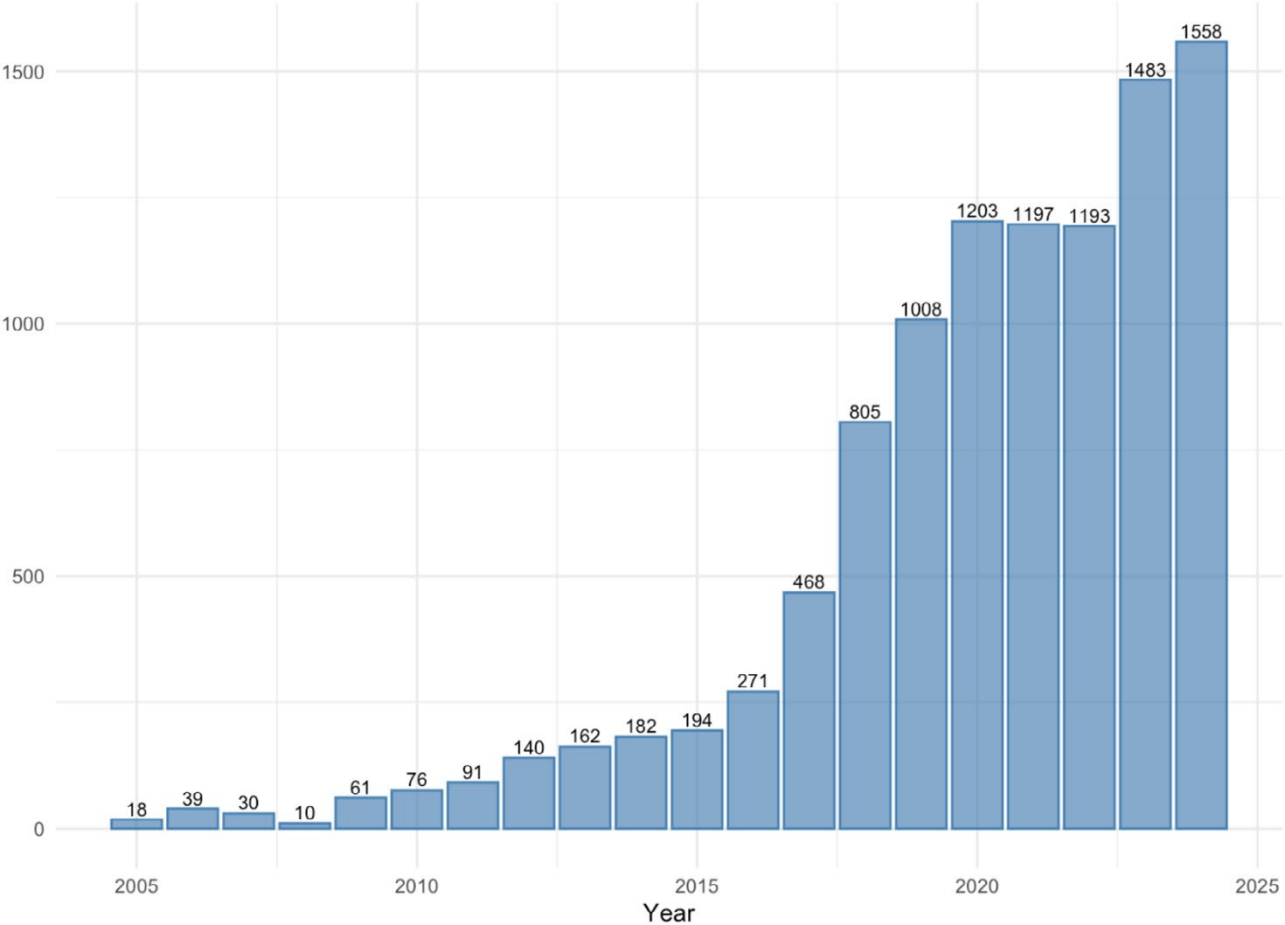
Number of anesthesiology and critical care podcast episodes published per year.

**FIGURE 3:**
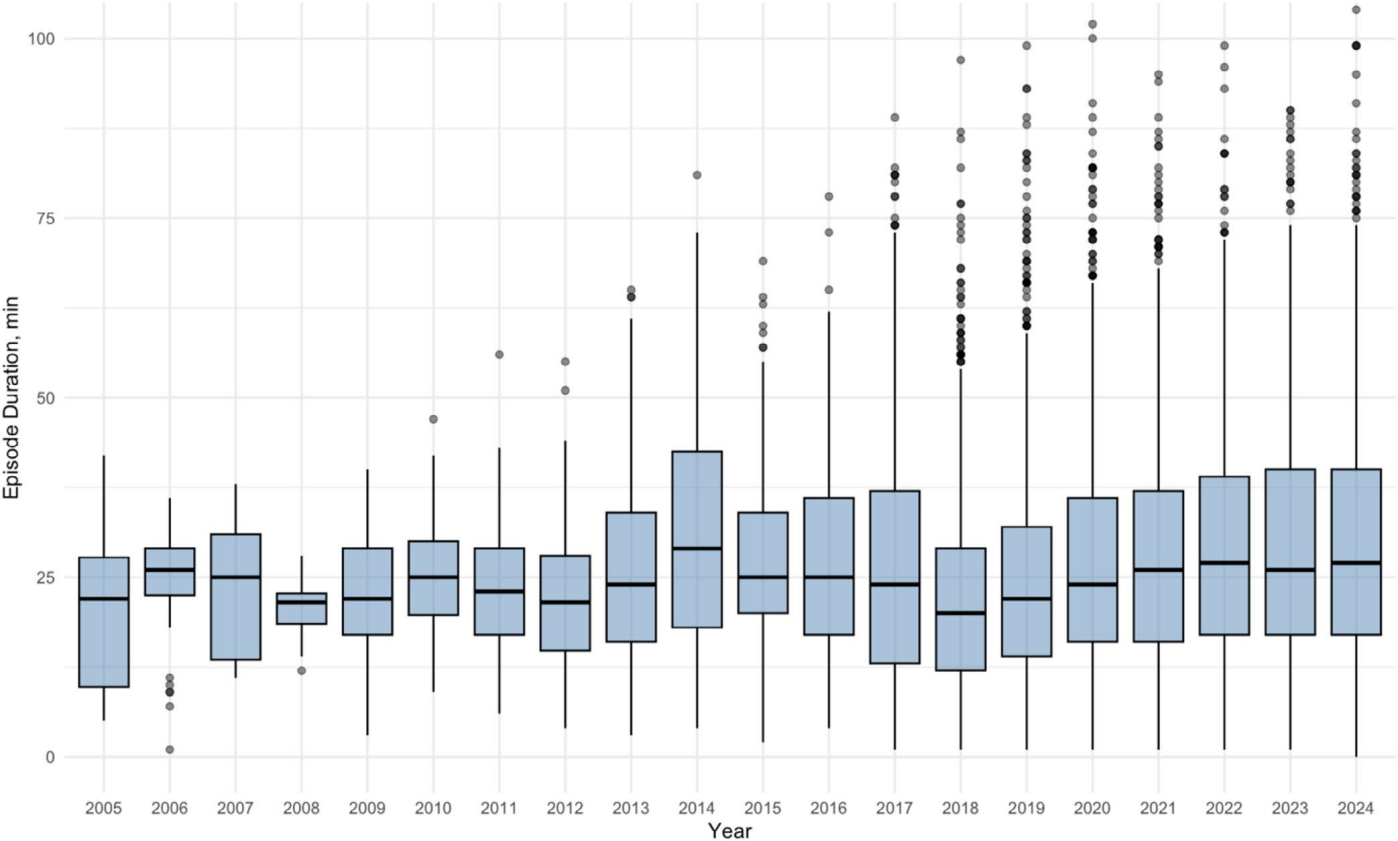
Median duration of anesthesiology and critical care podcast episodes by publication year. Box plots depict median (central line), interquartile range (IQR) (box), and whiskers extending to the most extreme data points within 1.5 × IQR; circles show outliers beyond this range.

### 3.3 Host Sex Distribution

Across 10,190 episodes, there was a total of 12,522 hosts: 8577 male (68.5%) and 3945 female (31.5%). Number of *individual* male and female hosts, per podcast, can be found in Table 1. The ratio of male to female hosts was 2.17:1. Most episodes (59.0%) featured exclusively male hosts, compared to 24.5% with exclusively female hosts, 10.7% with mixed‐sex hosts, and 5.7% with no defined host.

### 3.4 Guest Sex Distribution

The number of guests per episode ranged from 0 to 14. In total, there were 10,822 guests: 6940 male (64.1%) and 3882 female (35.9%); hence, the ratio of male to female guests was 1.79:1. A single guest was most common (52.2% of episodes), while only 2.7% of episodes had more than three guests. By episode composition, 27.1% had no guest, 10.0% had mixed‐sex guests, 20.3% had only female guest(s), and 42.5% had only male guest(s). Thus, episodes with only male guest(s) were more than twice as common as those with only female guest(s).

### 3.5 Association Between Host and Guest Sex

Episodes with exclusively male hosts were significantly more likely to feature only male guests (odds ratio [OR] 2.13 (95% CI 1.88–2.41), *p* < 0.001). Conversely, episodes with exclusively female hosts were significantly less likely to feature only male guests (OR 0.47 (95% CI 0.42–0.53), *p* < 0.001).

### 3.6 Temporal Changes in Sex‐Distribution of Podcast Hosts and Guests

Analysis using the “Male–Female Index” indicates an approximately linear decline in the proportion of male hosts and guests from 2010 onward (Figure 4). Between 2010 and 2024, the index decreased from 0.92 to 0.68 for hosts and from 0.77 to 0.59 for guests. Despite the observed decline, the 2024 index values remain above the parity threshold of 0.5, indicating that male representation continues to exceed female representation.

**FIGURE 4:**
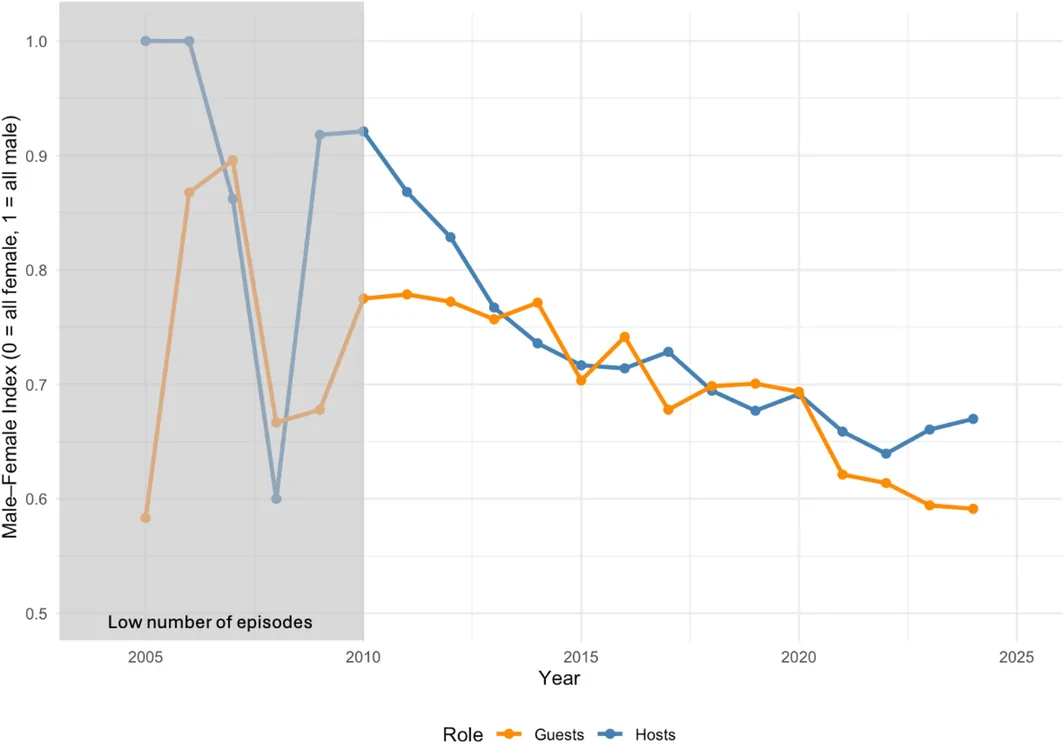
Temporal changes in sex‐distribution among podcast hosts and guests. The “Male–Female Index” is defined as the proportion of males relative to the total: Male(s)/male(s) + female(s), where 0 represents an entirely female sample and 1 an entirely male sample.

### 3.7 Subgroup Analysis: Sex Distribution by Podcast Country of Origin

Table S1 details the subgroup analyses of female representation by podcast country of origin. Focusing on the US‐based subgroup, which comprised 53.0% of all podcasts, women accounted for 27.9% of hosts (*n* = 2006) and 37.2% of guests (*n* = 2234). In Australia/New Zealand, the proportions of female hosts and guests were broadly similar. By contrast, podcasts from Great Britain featured a greater proportion of female hosts (40.7%), but a slightly lower proportion of female guests (33.9%).

### 3.8 Indicators of Quality and Review Processes

Use of show notes and supplemental data varied significantly between episodes. Among all 10,190 episodes, 42.0% included show notes (ranging from brief summaries to detailed outlines and comprehensive infographics), and 48.4% provided supplemental material (most commonly reference lists and article links). The percentage of episodes containing show notes and supplemental material per podcast is presented in table 1.

Analyses of variables related to peer‐review processes [10] revealed the following characteristics: 40.0% (*n* = 34) of podcasts produced (at least some) episodes linked to a publication, 50.6% (*n* = 43) featured episodes with three or more speakers, 21.2% (*n* = 18) of podcasts were journal‐affiliated, and 20.0% (*n* = 17) were university‐affiliated.

## 4 Discussion

### 4.1 Summary of Findings

In this systematic search and descriptive analysis of 85 anesthesiology and critical care podcasts (10,190 episodes), we found a significant male predominance among hosts (male to female ratio 2.17:1) and guests (1.79:1). Notably, episodes with exclusively male hosts were more than twice as likely (OR 2.13) to feature exclusively male guests. However, longitudinal analysis from 2010 to 2024 reveals a clear, near‐linear trend toward increased female participation as both hosts and guests. While a strong female–male imbalance persists, this shift marks measurable, ongoing progress toward greater diversity in the field. Additionally, our data highlight important aspects of quality assurance processes related to podcasts.

### 4.2 Sex Representation in Context: Workforce Benchmarks and Persistent Network Effects

Comparable sex imbalances have been documented in broader podcasting. A 2022 genre analysis (including 592 podcasts) found that female hosts constituted just 33.2% of hosts and were underrepresented in seven of eight popular genres, the exception being true crime [11]. The study also reported a pronounced female guest deficit (ratio of 1 female to 2.7 male guests) and found that female‐hosted shows featured significantly more female guests (45.3% vs. 17.9%). Importantly, beyond the scope of the present work, a striking underrepresentation of women of color was also found [11]. Consistent with our results, an analysis of three leading emergency medicine podcasts revealed a marked sex gap over a 10‐year period: 89.8% of hosts and 76.5% of guests were male [5]. This study also documented a positive temporal trend, with the proportion of female speakers rising from 9.1% to 23.1% between 2011 and 2021. Similarly, a recent detailed study focusing on 55 trauma surgery podcast episodes found that a majority was hosted by males (85%) [12].

When interpreting our findings, workforce demographics provide an essential benchmark. In the US, where the majority of analysed podcasts originate, female physicians constituted 26.5% of anesthesiologists and 28.5% of critical care physicians in 2023 [7]. Notably, the proportion of female guests in our US‐podcast sample (37.2%) exceeds this workforce baseline. This suggests that, far from underrepresenting women, the US‐based anesthesiology podcasting landscape is currently amplifying female voices relative to their presence in the specialty. This is an encouraging finding and indicates that podcast creators may be actively seeking female speakers. However, while workforce data are available for other countries, the number of podcasts originating from each individual country in our sample was too small to perform meaningful country‐specific comparisons. In regions with higher female workforce representation, such as the UK (38%) and Australia/New Zealand (36%) [13, 14], the gap between podcast representation and workforce availability may narrow further, but this remains to be confirmed with larger, country‐specific samples.

We emphasize that equitable representation should not be conflated with numerical parity. Given that women constitute 26.5% of US anesthesiologists, a 50% female speaker composition would not reflect the available pool and would be an unrealistic benchmark. Rather, we argue that representation should be assessed proportionally, that is, relative to workforce demographics. By this metric, the podcasting landscape is performing favorably. The more pressing concern raised by our data is not the aggregate number, but persistent network effects: episodes with male hosts remain significantly more likely to feature exclusively male guests, suggesting that sex similarity in speaker selection persists even as overall numbers improve.

Inequity in speaker representation is a consistently documented problem in the medical community [15, 16, 17, 18, 19, 20, 21, 22, 23, 24]. A cross‐sectional analysis of medical conferences across all specialties in Canada and the US found that, despite some progress, the proportion of female speakers remained disproportionately low, increasing from only 24.6% in 2007 to 34.1% in 2017, yet still falling far short of equitable representation [15]. Similarly, an analysis of five critical care conferences from 2010 to 2016 revealed a persistent gender imbalance: Men consistently dominated, representing 62%–70% of physician speakers, whereas women made up just 10%–19% [25].

Additionally, an examination of anesthesiology grand rounds found that female presenters constituted only 28.3% of all speakers, a rate notably lower than their representation as faculty (34%) and residents (36.7%) [26]. These data suggest that the sex‐based selection patterns we observed in podcasting reflect broader systemic patterns in academic medicine, rather than a phenomenon unique to the medium.

### 4.3 The Role of Visibility in Addressing Professional Inequity

Visibility is widely regarded as essential to reducing professional inequities, yet its effects are neither automatic nor uniform. Studies from diverse settings, including a well‐known intervention in Indian village councils, suggest that exposure to female leaders can gradually shift implicit biases [27]. However, the generalizability of such findings to medical podcasting is not assured. A recent study found that even after affirmative action policies, female professors consistently drew significantly smaller audiences than their male counterparts, indicating that representation alone does not guarantee recognition or influence [28]. In the context of our findings, this suggests that increasing the number of female podcast speakers, while important, may be insufficient if implicit biases continue to shape whose voices are sought out, amplified, and trusted. The persistent sex concordance we observed, whereby male hosts disproportionately feature male guests, may be a more direct manifestation of these biases.

This matters beyond the podcasting medium itself. When women perceive conferences or professional forums as exhibiting sexist attitudes, they report a greater intention to leave academic careers [29]. This is reflected in data showing that female physicians are more likely to depart from academic medicine within 5 years compared to their male counterparts, with rates of 38.3% and 32.4%, respectively [30].

The presence of women physicians in academic medicine is essential for providing mentorship, sponsorship, and positive role models [31]. Their visibility directly nurtures the next generation by offering guidance and support throughout training and career development. A lack of retention among women academic physicians thus carries significant opportunity costs, limiting access to diverse mentorship and reducing the pool of potential leaders.

We therefore argue that visibility efforts must be paired with intentional strategies to counter recognition bias, such as structured guest selection processes, transparent diversity metrics, and active efforts to diversify editorial and hosting teams. Ultimately, when women are seen and heard in prominent roles, and when their contributions are genuinely recognized, the environment becomes more inclusive, fostering a culture where diversity is valued and supported.

### 4.4 Quality Assurance and Its Relationship to Representation

As podcasts continue to grow in popularity and influence within medical education, the question of how quality is maintained warrants careful consideration, but also humility. The low barrier to entry is not a bug, but a feature; it enables clinicians with limited institutional support to share expertise, offer counter‐narratives, and reach global audiences without traditional gatekeeping. Imposing rigid peer‐review requirements would risk silencing these very voices. Moreover, quality assurance mechanisms and representation are not independent dimensions of podcast design. Structured selection processes, such as those associated with journals, universities, or formal peer‐review, may introduce systematic guest selection criteria that mitigate reliance on informal networks, which often perpetuate existing demographic imbalances. Conversely, the absence of such structures may allow implicit biases in speaker selection to operate unchecked. We therefore examined quality indicators not as a secondary theme, but as potential institutional levers that could influence and potentially rectify the representation patterns documented in this study.

In the present review, fewer than half of episodes contained show notes (42.0%) or supplemental material (48.4%). These proportions are relatively low despite using broad, inclusive definitions for both content types. Importantly, while not systematically assessed, quality and comprehensiveness of show notes and supplemental material varied vastly. While there are extraordinary examples of infographics, visual abstract summaries, lists of pitfalls and pearls, detailed outlines including quizzes and take‐home‐points, most supplemental material was relatively limited. According to metrics defined in a previous attempt to assess rates of peer‐review in anesthesiology podcasts (“…created in the context of a publication, presence of three or more speakers, grand rounds, and association with a journal or university”) [10], 72.9% of podcasts fulfilled at least one criteria. Although podcast websites often feature chat forums that facilitate rapid feedback, the heterogeneous nature of these platforms complicated systematic data collection. Despite this, reviewers noted multiple instances of post‐publication corrections, highlighting a key strength of podcasts compared to traditional publishing channels [6].

Rather than advocating for mandatory peer review, we suggest a more differentiated framework. Podcasts associated with academic institutions or journals may reasonably adopt structured review processes, while independent podcasts might prioritize transparent disclosure of content sources and potential conflicts of interest. The heterogeneity of current practices suggests substantial room for incremental improvement without compromising accessibility. Ultimately, we call for transparency, enabling listeners to make informed judgments about content credibility, much as they would with any other educational resource.

### 4.5 Strengths and Limitations

This is the first systematic search and descriptive analysis of anesthesiology and critical care podcasts and, to our knowledge, the largest review of medical podcasts to date, analyzing over 10,000 episodes across 85 podcasts. Its primary strength is the substantial sample size. To mitigate single reviewer error, 10% of the data was independently double‐extracted.

Several limitations are noted. First, data extraction was performed by multiple reviewers to manage the scale of the task, a necessary approach given the project's scope. This may introduce inter‐rater variability, despite team meetings held to synchronize procedures. Second, our analysis categorizes speaker sex as a binary variable (male/female) using proxies such as names, voice, and pronouns. We recognize that pronouns denote gender identity rather than biological sex, and that our binary classification does not capture intersex, non‐binary, or self‐determined gender identities, a conceptual limitation that risks perpetuating homogenizing norms. Third, although we used multiple identifiers, there remains a risk of misclassification. Fourth, our inclusion criterion restricting analysis to physician‐hosted podcasts may have introduced selection bias; podcasts hosted by nurses or multidisciplinary teams could exhibit different sex compositions. Fifth, we did not systematically assess podcast funding sources, commercial status, advertising, or sponsorship, all of which may influence content, speaker selection, and editorial independence. Sixth, our analysis focused exclusively on sex representation, and we acknowledge the absence of data on racial and ethnic diversity as a limitation. However, reliably categorizing speaker race or ethnicity from audio, names, or show notes is fraught with methodological challenges.

Finally, the geographic distribution of our sample, predominantly US‐based, limits the direct generalizability of our findings to regions with different workforce demographics. While workforce data are available for other countries, the number of podcasts originating from each individual country in our sample was too small, with the exceptions of Australia/New Zealand and Great Britain, to support meaningful country‐specific comparisons. Relatedly, our findings must be interpreted alongside evidence that male sex is predictive of podcast use among anesthesiologists [6]. If female clinicians are less likely to consume this medium, then the underrepresentation of female voices both stems from and potentially perpetuates a feedback loop: male‐dominated content may appeal less to female listeners, while lower female listenership may reduce incentives for podcasters to recruit female speakers. This bidirectional relationship complicates simple interpretations of our data.

## 5 Conclusions

This review documents that while a sex imbalance persists in anesthesiology and critical care podcasting, female participation actually appears to exceed workforce demographics, a positive trend that has strengthened linearly from 2010 to 2024. However, persistent sex similarity in speaker selection suggests that biases remain. We propose three actionable pathways for stakeholders: (1) Podcast creators and hosts could adopt structured guest selection processes to reduce reliance on informal networks; (2) professional societies could develop voluntary guidelines for inclusive representation and transparent quality indicators; and (3) academic departments could recognize podcast contributions as scholarly activity, thereby incentivizing diverse participation. By aligning these efforts with the medium's inherent accessibility, the community can accelerate progress toward both equity and educational quality in medical knowledge dissemination.

## Author Contributions

**P.S.:** conceptualization, methodology, investigation, writing – original draft preparation; **S.A.:** methodology, investigation, writing – review and editing; **R.N.K.:** investigation, writing – review and editing; **A.R.:** writing – review and editing, formal analysis, visualization; **A.N.:** investigation, writing – review and editing; **N.D.:** investigation, writing – review and editing; **S.Å.F.:** investigation, writing – review and editing; **J.H.:** investigation, writing – review and editing; **M.F.B.:** conceptualization, methodology, formal analysis, investigation, resources, writing – original draft, supervision, project administration.

## Funding

The authors have nothing to report.

## Conflicts of Interest

The authors declare no conflicts of interest.

## Supporting information


**Table S1:** Subgroup analyses of female representation by podcast country of origin.

## Data Availability

The data that support the findings of this study are available from the corresponding author upon reasonable request.
