# Supplementary material for: Female Representation and Quality Indicators in Anesthesiology and Critical Care Podcasts
Source: Acta Anaesthesiol Scand. 2026 Oct 14;70(10):e70356. doi: 10.1111/aas.70356 (PMC13669520; doi:10.1111/aas.70356)
Supplement: Supplementary file 1 [file AAS-70-0-s001.docx]

| Country | Podcasts | Proportion female hosts | Proportion female guests |
| --- | --- | --- | --- |
| United States | 45 | 27.9% (2006 / 7184) | 37.2% (2236 / 6012) |
| Australia / New Zealand | 16 | 33.1% (489 / 1478) | 37.9% (243 / 642) |
| Great Britain | 11 | 40.7% (1310 / 3215) | 33.9% (1117 / 3291) |
| International | 5 | 34.7% (100 / 288) | 37.9% (111 / 293) |
| Canada | 3 | 8.3% (13 / 156) | 35.3% (59 / 167) |
| India | 2 | 62.9% (22 / 35) | 25.6% (20 / 78) |
| Ireland | 2 | 0% (0 / 152) | 28.7% (96 / 334) |
| Singapore | 1 | 35.7% (5 / 14) | 0% (0 / 5) |
